# Case of nasopharyngeal tuberculosis complicated with cervical lymph node and pulmonary tuberculosis

**DOI:** 10.1515/biol-2022-1041

**Published:** 2025-04-14

**Authors:** Jialin Cao, Hui Gan, Jian-Hui Wu, Yue Yuan, Fei Ye

**Affiliations:** The First Clinical Medical College of Guangdong Medical University, Zhanjiang, Guangdong 524000, P. R. China; The First Affiliated Hospital of Guangzhou Medical University, 510180, Guangzhou, Guangdong, P. R. China; The Department of Otolaryngology in the Zhongshan City People’s Hospital, Zhongshan, Guangdong, P. R. China

**Keywords:** nasopharyngeal tuberculosis, cervical lymph node tuberculosis, diagnosis

## Abstract

Multiple cases of extrapulmonary tuberculosis (TB) combined with pulmonary TB are commonly encountered in clinical practice. Nasopharyngeal TB is often ignored because of its hidden location and nonspecific manifestations. A correlation between cervical lymph node and nasopharyngeal TB has been observed; however, reports of cervical lymph node TB complicated with nasopharyngeal TB are rare, potentially leading to missed diagnosis. Herein, we present a case of nasopharyngeal TB combined with cervical lymph node and pulmonary TB. Radiographic findings consistently suggested TB, although multiple smear tests’ results were negative. After 3 months of diagnostic anti-TB treatment, the nasopharyngeal TB completely disappeared, and the swollen cervical lymph nodes subsided after 1 year of treatment. This case report deepens our understanding of nasopharyngeal TB and emphasizes the need for nasal endoscopy in patients with cervical lymph node TB.

## 1 Introduction

China has the third largest tuberculosis (TB) burden in the world, accounting for approximately 7.1% of new TB cases globally in 2022 [1]. In recent years, the overall TB incidence has declined in China [2]. Although the overall TB incidence has declined, extrapulmonary TB (EPTB) has been increasing in certain regions [3]. EPTB accounts for approximately 20% of all TB cases [4]. Nasopharyngeal TB is a rare form of EPTB and reported to constitute less than 1% of respiratory TB [5]. Patients with nasopharyngeal TB, cervical lymph node TB, and pulmonary TB are even rarer. These patients often present nonspecific symptoms, leading to nasopharyngeal TB being frequently overlooked in clinical practice.

The main diagnostic techniques for TB include culture, microscopic acid-fast bacillus detection, histopathology, and polymerase chain reaction (PCR) [6]. However, not every patient has a positive result. The sensitivities of sputum smear microscopy, sputum culture, and fluorescence quantitative PCR are reported to be 33.3, 44.4, and 33.3%, respectively [7]. With the advancement of molecular diagnostic technology, the Xpert Ultra test has greatly improved the accuracy of TB detection and is the molecular rapid diagnostic test recommended by the World Health Organization (WHO) [8]. The complete diagnosis and treatment of a patient with nasopharyngeal TB combined with cervical lymph node and pulmonary TB, followed up for 1 year, were reported. The diagnosis, differential diagnosis, and therapeutic approaches were discussed, and the relevant literature was reviewed.

## 2 Case report

**Figure 1:**
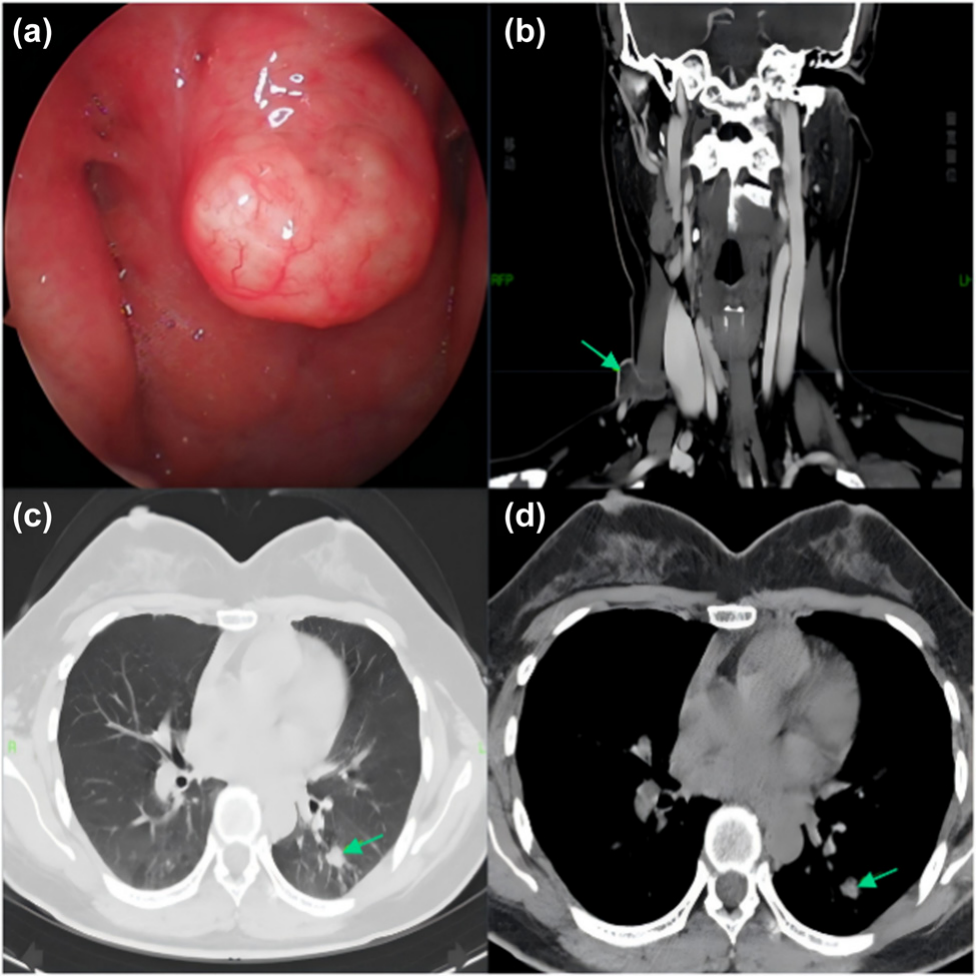
Initial findings: (a) smooth mass on the posterior wall of the nasopharyngeal roof under nasal endoscopy, without the accumulation of bilateral pharyngeal recesses; (b) annularly enhanced lymph nodes were observed in the region IV of the right neck, with annular enhanced foci under the adjacent skin with the measured size being about 22 mm × 14 mm, and sinus communication observed between the two; (c and d) large solid nodules (green arrow) in the left lower lung, suggesting secondary TB.

**Figure 2:**
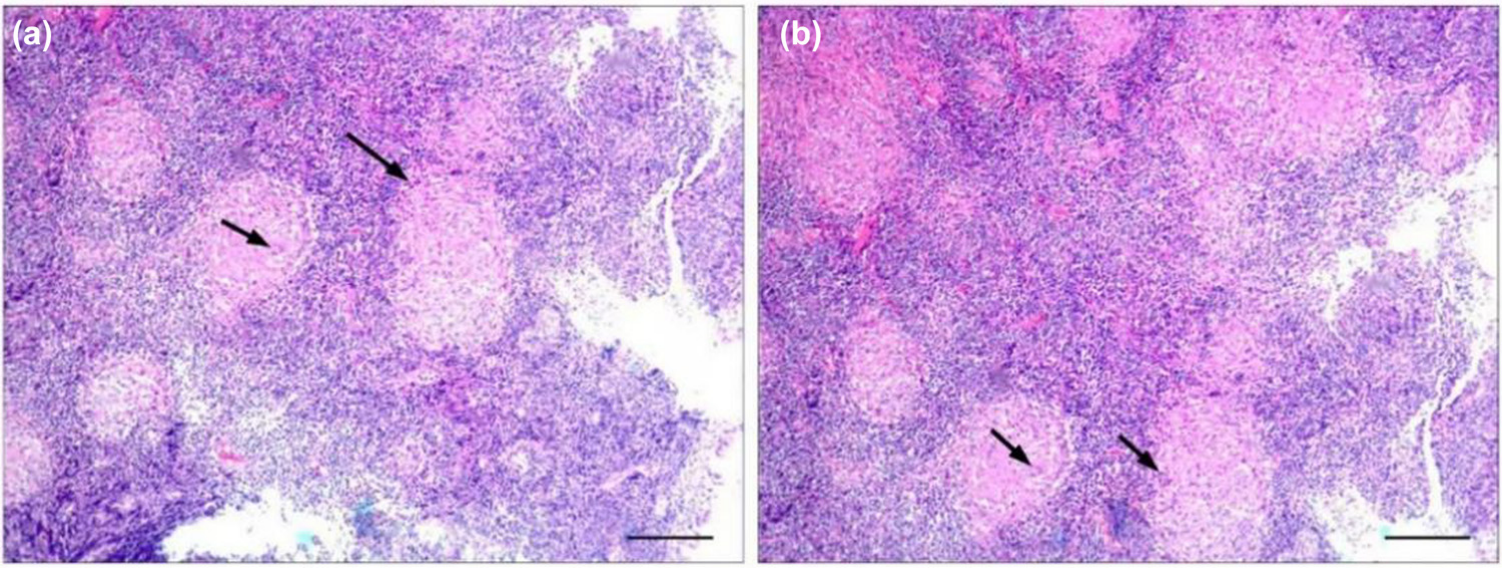
HE staining image of nasopharyngeal pathological tissue under 40× microscope: (a and b) local necrosis of the nasopharynx, surrounded by epithelioid cells, infiltration of lymphocytes, and plasma cells, was observed under pathological microscope, which was consistent with granulomatous inflammation. Arrows indicate multinuclear giant cells. The scale in the lower-right corner of the picture is 200 µm.

A 37-year-old woman presented to the Otolaryngology Department of Head and Neck Surgery on June 9, 2022, with a right neck mass that had persisted for half a month. She did not show any positive symptoms, such as nasal obstruction, hearing impairment, cough, sputum, night sweats, or weight loss, and had no history of TB contact. Physical examination revealed local swelling, swelling of the right neck, rising skin temperature, tenderness, and a fluctuating sensation. The nasopharyngeal masses were observed using an indirect laryngoscope. Nasopharyngeal cancer is the first consideration because of its high incidence in the local area. A nasal endoscopy was performed on the patient. The nasal endoscopic findings showed smooth round masses on the posterior wall of the nasopharyngeal roof, without the accumulation of bilateral pharyngeal recesses (Figure 1a). To rule out neck and chest tumors, the patient was followed by enhanced cervical computed tomography (CT) and plain scan CT of the lungs. Enhanced cervical CT showed a circular enhanced lymph node in the right neck region IV, and a circular enhanced focus could be seen under the adjacent skin with the measured size being about 22 mm × 14 mm; and large solid nodules were seen in the left lower lung (Figure 1b–d). To clarify the diagnosis, pathological biopsy was performed using a rigid nasal endoscope at 0° under local anesthesia. After acid-fast staining did not show a positive result, hematoxylin–eosin (HE) staining was performed on the pathological specimen after surgery, and multinucleated giant cells were observed at a magnification of 40× (Figure 2a and b, indicated by the black arrow). Outpatient diagnoses included nasopharyngeal, lymph node (right neck), and pulmonary TB.

**Figure 3:**
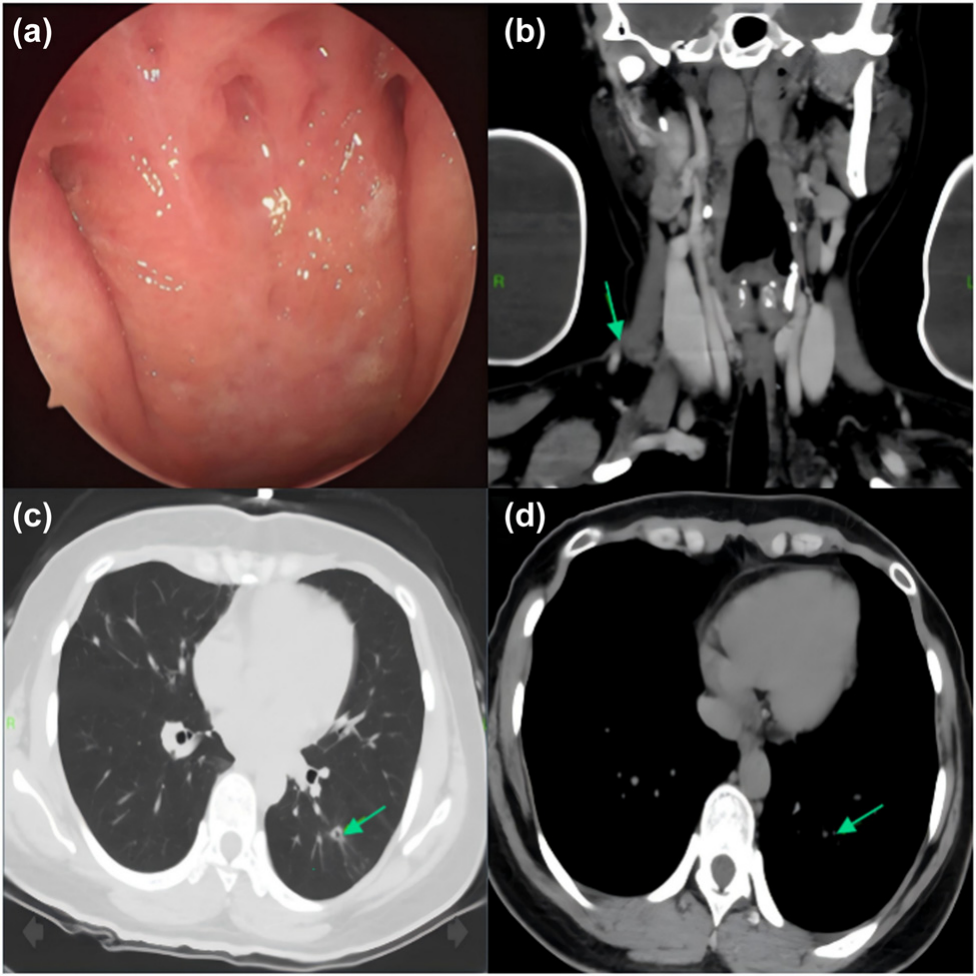
Manifestations after 3 months of treatment: (a) nasopharyngeal masses disappeared under nasal endoscopy; (b) annularly enhanced lymph nodes and adjacent subcutaneous tuberculous abscesses in the right neck region IV were reduced, and the measured size was about 15 mm × 4 mm; (c and d) left-lower pulmonary nodules were further absorbed, with small cavities observed inside.

**Figure 4:**
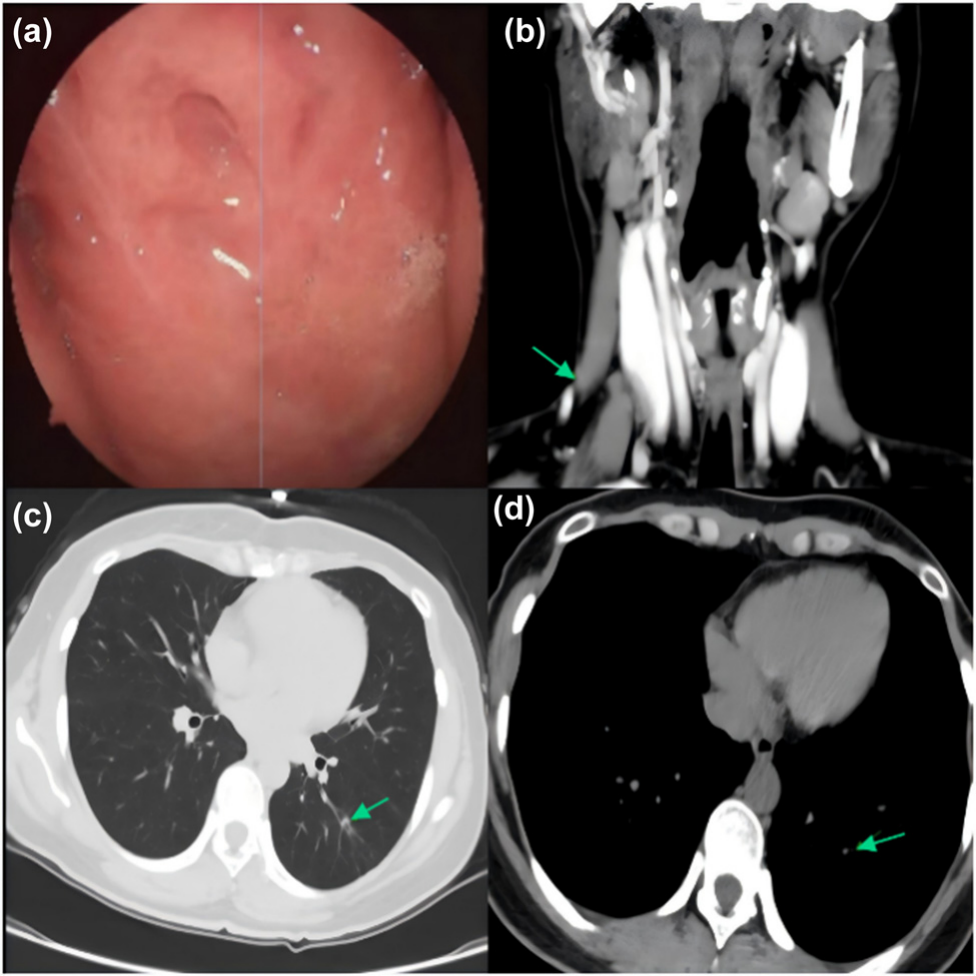
Manifestations after 12 months of treatment: (a) scar hyperplasia was observed locally in the nasopharynx without recurrence; (b) original tuberculous abscess had mostly dissipated, surface skin was slightly thickened, and (c and d) the left-lower lung nodule was further reduced.


After multiple sputum smear examination and *Mycobacterium* TB culture failed to detect acid-fast bacilli, it was recommended to start diagnostic anti-TB therapy early following a respiratory consultation, and start systemic anti-TB therapy using the 2HRZE/10HRE regimen, i.e., 2 months of intensification (isoniazid [INH] 300 mg, rifampicin [RFP] 600 mg, pyrazinamide [PZA] 1,500 mg, ethambutol [EMB] 1,000 mg, all once daily) followed by 10 months of consolidation (stopping PZA while maintaining the original dose of other drugs). During the treatment, the patient did not experience significant physical discomfort, such as gastrointestinal reactions, fatigue, loss of appetite, and blurred vision. After 3 months of treatment, the nasopharyngeal masses were found to have largely disappeared upon re-examination with nasal endoscopy (Figure 3a). Then, neck enhanced CT and chest plain CT were performed, showing that the enlarged lymph nodes in the neck had not shrunk significantly and the diameter was approximately 6 mm; however, the area of the subcutaneous abscess had significantly reduced with the measured size being about 15 mm × 4 mm, and the size of the pulmonary nodule in the lower left lung was remarkably reduced with small cavities observed inside (Figure 3b–d). After 12 months of follow-up, the nasopharyngeal mass had disappeared completely (Figure 4a), with no signs of recurrence. Enhanced neck and chest CT revealed that the original tuberculous abscess in the right neck had dissipated, swollen lymph nodes in the neck had subsided, and secondary pulmonary TB in the left lung was smaller than before, with no related symptoms or evidence of pulmonary TB activity (Figure 4b–d).

**Informed consent:** Informed consent was obtained from all individuals included in this study.
**Ethical approval:** The research related to human use complied with all relevant national regulations and institutional policies in accordance with the tenets of the Helsinki Declaration and was approved by the authors’ institutional review board or equivalent committee.

## 3 Discussion

Nasopharyngeal TB is a rare form of EPTB. The clinical manifestations are non-specific, often presenting mild or no discomfort, and may include symptoms such as persistent sore throat, dysphagia, hoarseness, nasal congestion, and a sensation of a lump in the throat [9]. When patients present to the otolaryngology department with similar nose and throat symptoms, diagnosis can be challenging for doctors. Nasopharyngeal TB can be combined with a variety of EPTB, and it may be missed diagnosed when cervical lymph node TB is a manifestation. Among the 50 cases of nasopharyngeal TB reported by Jian et al. [10], 60% cases were cervical lymph node TB. Many patients with cervical lymph node TB rarely show signs of nasopharyngeal TB, and the lack of nasal endoscopy during their disease progression may lead to a certain extent of missed diagnoses [11,12].

In this case report, the patient also presented with lymph node enlargement combined with abscess formation. For otolaryngology specialists, nasal endoscopy has been frequently used in this profession. When receiving patients with cervical lymph node enlargement, nasal endoscopy is almost one of the items to be checked, especially in the region where the case belongs to the high incidence of nasopharyngeal cancer. When patients are referred to other departments, nasopharyngeal lesions may be overlooked. Therefore, nasal endoscopy is necessary for patients with cervical lymph node TB.

Once diagnosed, early standardized anti-TB drug treatment can achieve good results. No cases of drug resistance or treatment failures were observed. The treatment of EPTB is similar to that of pulmonary TB [13], with the shortest treatment time being 6 months. Isoniazid (H), rifampicin (R), pyrazinamide (Z), ethambutol (E), and other drugs are used in combination to inhibit the synthesis of DNA, RNA, or cell wall of TB bacteria to inhibit their reproduction and bactericidal effects. Waldron et al. [14] advocated the standard treatment of INH, RFP, and PZA for at least 6 months, and the addition of streptomycin within the first 2–3 months. Chiesa Estomba et al. [15] preferred 2 months of INH, RFP, EBM, and PZA, followed by 4–7 months of INH and RFP. Data show that the average treatment time for cervical lymph node TB is 14.91 ± 7.03 months [16]. Some studies report that the cure rate for cervical lymph node TB can reach 63.3% with a treatment period of 6 months [17]. In addition, the WHO strongly recommends a 6-month treatment program for patients with drug-sensitive TB, according to the WHO guidelines for drug-sensitive anti-TB treatment and patient care. In this case, the nasopharyngeal lesions basically subsided after 3 months of conventional anti-TB treatment, whereas the cervical lymph node disappeared after 12 months of treatment for cervical lymph node TB. This case and a review of the relevant literature indicate that the treatment period of nasopharyngeal TB is significantly shorter than that of cervical lymph node TB. Whether this significant therapeutic effect is related to local tissue bacterial load requires further investigation. This study has some limitations. Although the imaging in this case pointed to TB infection and the effectiveness of anti-TB therapy, etiological evidence is lacking in the diagnosis and treatment process.

## 4 Conclusion

Patients with nasopharyngeal TB combined with cervical lymph node TB occasionally present in clinical practice. However, owing to its hidden location and lack of specificity in clinical manifestations, it is often ignored, and cervical lymph node TB is frequently the primary diagnosis. Therefore, nasal endoscopy should be performed in patients with cervical lymph node TB to reduce missed diagnoses, particularly in areas with a high incidence of nasopharyngeal cancer. In addition, this case suggests that the treatment period of nasopharyngeal TB is significantly shorter than that of cervical lymph node TB.
